# Can Broader Diffusion of Value-Based Insurance Design Increase Benefits from US Health Care without Increasing Costs? Evidence from a Computer Simulation Model

**DOI:** 10.1371/journal.pmed.1000234

**Published:** 2010-02-16

**Authors:** R. Scott Braithwaite, Cynthia Omokaro, Amy C. Justice, Kimberly Nucifora, Mark S. Roberts

**Affiliations:** 1 Section of Value and Comparative Effectiveness, New York University School of Medicine, New York, New York, United States of America; 2 Albany Medical College, Albany, New York, United States of America; 3 General Internal Medicine, Yale University Schools of Medicine and Public Health, New Haven, Connecticut, United States of America; 4 Section of Decision Sciences and Clinical Systems Modeling, University of Pittsburgh School of Medicine, Pittsburgh, Pennsylvania, United States of America; 5 Department of Health Policy and Management, University of Pittsburgh Graduate School of Public Health, Pittsburgh, Pennsylvania, United States of America; Harvard School of Public Health, United States of America

## Abstract

**Background:**

Evidence suggests that cost sharing (i.e.,copayments and deductibles) decreases health expenditures but also reduces essential care. Value-based insurance design (VBID) has been proposed to encourage essential care while controlling health expenditures. Our objective was to estimate the impact of broader diffusion of VBID on US health care benefits and costs.

**Methods and Findings:**

We used a published computer simulation of costs and life expectancy gains from US health care to estimate the impact of broader diffusion of VBID. Two scenarios were analyzed: (1) applying VBID solely to pharmacy benefits and (2) applying VBID to both pharmacy benefits and other health care services (e.g., devices). We assumed that cost sharing would be eliminated for high-value services (<$100,000 per life-year), would remain unchanged for intermediate- or unknown-value services ($100,000–$300,000 per life-year or unknown), and would be increased for low-value services (>$300,000 per life-year). All costs are provided in 2003 US dollars. Our simulation estimated that approximately 60% of health expenditures in the US are spent on low-value services, 20% are spent on intermediate-value services, and 20% are spent on high-value services. Correspondingly, the vast majority (80%) of health expenditures would have cost sharing that is impacted by VBID. With prevailing patterns of cost sharing, health care conferred 4.70 life-years at a per-capita annual expenditure of US$5,688. Broader diffusion of VBID to pharmaceuticals increased the benefit conferred by health care by 0.03 to 0.05 additional life-years, without increasing costs and without increasing out-of-pocket payments. Broader diffusion of VBID to other health care services could increase the benefit conferred by health care by 0.24 to 0.44 additional life-years, also without increasing costs and without increasing overall out-of-pocket payments. Among those without health insurance, using cost saving from VBID to subsidize insurance coverage would increase the benefit conferred by health care by 1.21 life-years, a 31% increase.

**Conclusion:**

Broader diffusion of VBID may amplify benefits from US health care without increasing health expenditures.

*Please see later in the article for the Editors' Summary*

## Abstract 2

Using a computer simulation based on US data, R. Scott Braithwaite and colleagues calculate the benefits of value-based insurance design, in which patients pay less for highly cost-effective services.

## Editors' Summary

**Background:**

More money is spent per person on health care in the US than in any other country. US health care expenditure accounts for 16.2% of the gross domestic product and this figure is rising. Indeed, the increase in health care costs is outstripping the economy's growth rate. Consequently, US policy makers and providers of health insurance—health care in the US is largely provided by the private sector and is paid for through private health insurance or through government programs such as Medicare and Medicaid—are looking for better ways to control health expenditures. Although some health care cost reductions can be achieved by increasing efficiency, controlling the quantity of health care consumed is an essential component of strategies designed to reduce health expenditures. These strategies can target health care providers (for example, by requiring primary care physicians to provide referrals before their patients' insurance provides cover for specialist care) or can target consumers, often through cost sharing. Nowadays, most insurance plans include several tiers of cost sharing in which patients pay a larger proportion of the costs of expensive interventions than of cheap interventions.

**Why Was This Study Done?:**

Cost sharing decreases health expenditure but it can also reduce demand for essential care and thus reduce the quality of care. Consequently, some experts have proposed value-based insurance design (VBID), an approach in which the amount of cost sharing is set according to the “value” of an intervention rather than its cost. The value of an intervention is defined as the ratio of the additional benefits to the additional costs of the intervention when compared to the next best alternative intervention. Under VBID, cost sharing could be waived for office visits necessary to control blood pressure in people with diabetes, which deliver high-value care, but could be increased for high-tech scans for dementia, which deliver low-value care. VBID has been adopted by several private health insurance schemes and its core principal is endorsed by US policy makers. However, it is unclear whether wider use of VBID is warranted. In this study, the researchers use a computer simulation of the US health care system to estimate the impact of broader diffusion of VBID on US health care benefits and costs.

**What Did the Researchers Do and Find?:**

The researchers used their computer simulation to estimate the impact of applying VBID to cost sharing for drugs alone and to cost sharing for drugs, procedures, and other health care services for one million hypothetical US patients. In their simulation, the researchers eliminated cost sharing for services that cost less than US$100,000 per life-year gained (high-value services) and increased cost-sharing for services that cost more than US$300,000 per life-year gained (low-value services); cost-sharing remained unchanged for intermediate- or unknown-value services. With the current pattern of cost sharing, 60% of health expenditure is spent on low-value services and health care increases life expectancy by 4.70 years for an annual per person expenditure of US$5,688, the researchers report. With widespread application of VBID to cost sharing for drugs alone, health care increased life expectancy by an additional 0.03 to 0.05 years without increasing costs. With widespread application of VBID to cost sharing for other health care services, health care increased life expectancy by a further 0.24 to 0.44 years without additional costs. Finally, if the costs saved by applying VBID were used to subsidize insurance for the 15% of the US population currently without health insurance, the benefit conferred by health care among these people would increase by 1.21 life-years.

**What Do These Findings Mean?:**

The findings of this study depend on the many assumptions included in the computer simulation, which, although complex, is a greatly simplified representation of the US health care system. Nevertheless, these findings suggest that if VBID were used more widely within the US health care system to encourage the use of high-value services, it might be possible to amplify the benefits from US health care without increasing health expenditures. Importantly, the money saved by VBID could be used to help fund universal insurance, a central aim of US health care reform. More research is needed, however, to determine the value of various health care interventions and to investigate whether other ways of linking value to cost sharing might yield even better gains in life expectancy at little or no additional cost.

**Additional Information:**

Please access these Web sites via the online version of this summary at http://dx.doi.org/10.1371/journal.pmed.1000234.

- Wikipedia has a page on health care in the United States (note that Wikipedia is a free online encyclopedia that anyone can edit; available in several languages)
- Families USA works to promote high-quality affordable health care for all Americans and provides information about all aspects of US health care and about US health care reforms
- The US Centers for Medicare and Medicaid provides information on the major government health insurance programs and on US national health expenditure statistics

## Introduction

Health plans, employers, and policymakers are looking for more effective approaches to control health expenditures. Reductions in health expenditures can arise from lowering either the costs or the quantities of health services [1]. While new initiatives have the potential to lower costs (e.g., increasing efficiency with health information technology), controlling quantity is likely to remain an essential component of any expenditure-control strategy. Strategies to reduce health service quantity in the US have typically targeted providers (e.g., preauthorization review) more than consumers (e.g., cost sharing). However, because health care costs continue to increase beyond the US economy's growth rate and targeting providers is often expensive and inefficient, increasing attention is focusing on approaches to lower consumer demand for health services, such as cost sharing.

Accordingly, cost sharing has become a ubiquitous feature of the US health care landscape. Nearly three-fourths of workers with employer-subsidized insurance enroll in plans with three or more cost sharing tiers [2], and the highest tiers have copayment rates averaging 36% [3]. While cost sharing is an effective way of decreasing health expenditures, it may lower demand for essential care and may lead to adverse outcomes, and therefore may reduce quality of care [2],[4]–[6]. For this reason, some have proposed the idea of value-based insurance design (VBID), which varies the amount of cost sharing according to either the incremental benefits of health services [7],[8] or to their “value,” as defined by the ratio of incremental benefits to incremental costs [9]. That way, rather than assigning a drug to a cost sharing tier based on its cost, VBID would assign it based on its value. For example, cost sharing could be waived for office visits and procedures necessary for blood pressure control or lipid reduction in diabetics, which deliver high-value care, but cost sharing could be increased for positron emission technology scans for dementia, which deliver low-value care [10]. Variants of VBID have been adopted by multiple employers, and its core principle—adjusting patient cost sharing to promote high-value care and discourage low-value care—has been endorsed by the Director of the Department of Health and Human Services Office of Health Reform [11].

**Figure 1:**
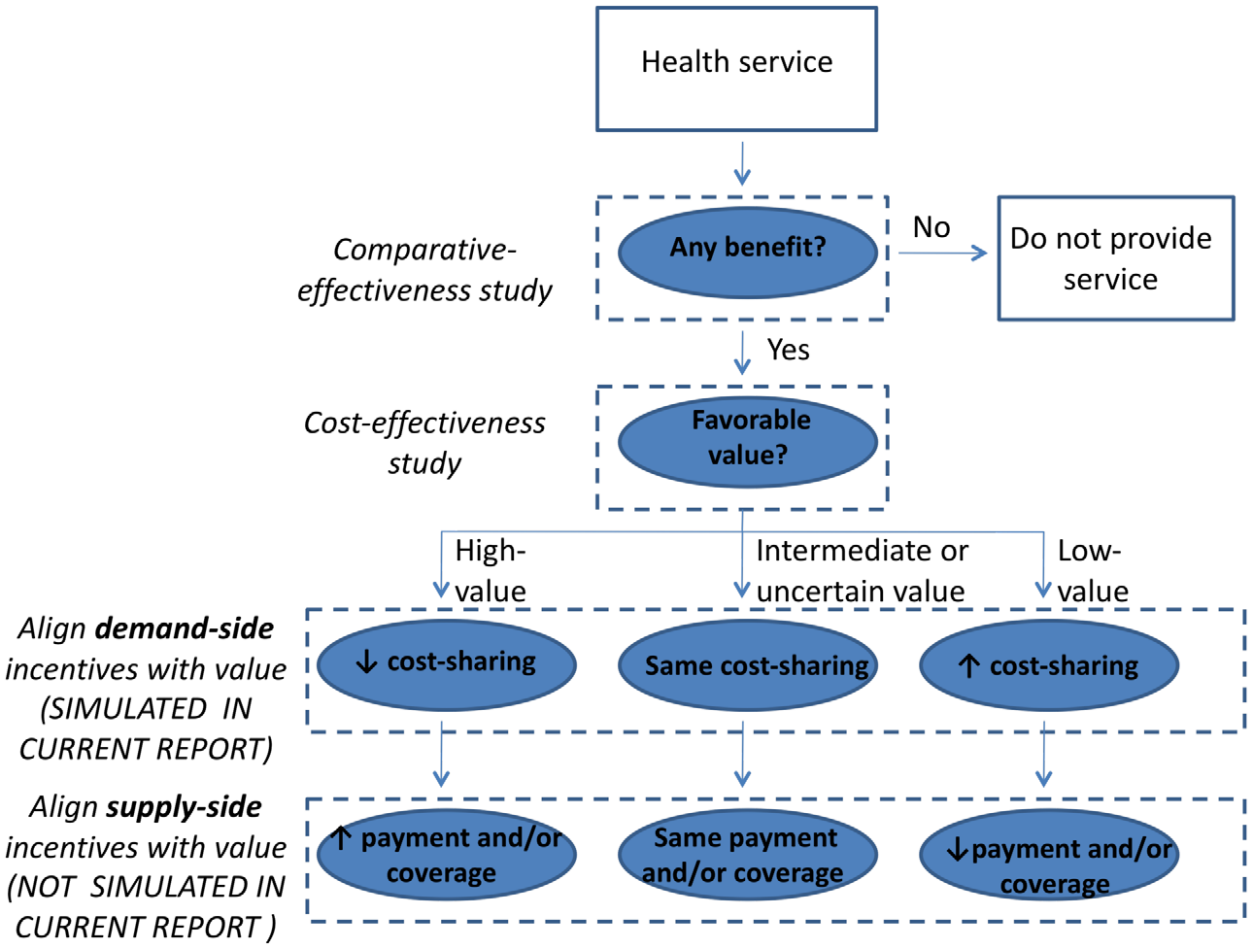
General framework for aligning health care incentives with value. Comparative effectiveness provides information about the incremental benefits and costs of a particular health service. This information is needed for assessing value, typically defined as the ratio of added benefits to added costs. Aligning demand-side incentives with value preserves consumer choice and avoids supply-side restrictions in payment and coverage. This process may proceed simultaneously for distinct patient subgroups that may each benefit from the intervention. Only demand-side incentives are modeled in the current report.

Pilot data suggest that VBID is feasible [5],[8],[12], successfully modulating the utilization of statins and other common drugs. While the rationale of VBID may be compelling, it is unclear whether broader diffusion of VBID is warranted. We used our validated computer simulation of the US health care system [13] to ask whether diffusion of VBID to other US health care settings (e.g., Centers for Medicare & Medicaid Services) could have a beneficial impact on health care costs and benefits. Because VBID is one among many possible frameworks for aligning health care incentives with value (Figure 1), this analysis may constitute one piece in the broader puzzle of how to use incentives systematically to encourage high-value care and to discourage low-value care.

## Methods

We evaluated two groups of scenarios involving broader diffusion of VBID. In the first group of scenarios, because cost sharing is a common attribute of medication coverage, we examined the effect of applying VBID to pharmacy benefits for all persons with health insurance in the US. In the second group of scenarios, we assumed that broader diffusion of VBID extends not only to pharmacy benefits, but also to other health care services (e.g., devices, procedures, etc.). Our rationale for performing this second, more hypothetical group of scenarios is that value assessment methods in other countries (e.g., UK, Canada, Australia, Germany) use the same tools for assessing the value of non-pharmaceutical services that they use for assessing the value of pharmaceuticals [14], and there is no theoretical rationale for using distinct methods. Therefore, VBID principles have the potential to be applied more broadly across health care services in the US. We define “cost sharing” as any copayment or deductible that is linked to a particular health service. Therefore, this definition does not include other types of payments (e.g., patients' share of insurance premium) or the indirect effects of employer health expenses on wages.

Within each of these two groups of scenarios, we analyzed the three following alternative design specifications (“strategies”) for VBID.

### 

#### Strategy 1

Do not require VBID implementation to be cost-neutral (no cost offset). Reduce cost sharing for high-value services to increase their demand, and do not change cost sharing for intermediate-value or low-value services.

#### Strategy 2

Require VBID implementation to be cost-neutral, without any intended impact on uninsurance (cost-offset value-based insurance design [COVID] without subsidy for uninsured). Reduce cost sharing for high-value services to increase their demand, do not change cost sharing for intermediate value services, and increase cost sharing for low-value services, to the extent necessary to offset additional costs from increasing demand for high-value services. We evaluated budget-neutrality from (A) a societal perspective (assuming that overall health expenditures should remain unchanged), (B) a payer's perspective (assuming that health plan expenditures should remain unchanged), and (C) a patient's perspective (assuming that out of pocket costs should remain unchanged).

#### Strategy 3

Require VBID to be cost-neutral, using a surplus obtained from lowering demand on low-value services to offset additional costs from increasing demand for high-value services and to subsidize expansion of health insurance coverage (COVID with subsidy for uninsured). Similarly to strategies 1 and 2, this alternative would reduce cost sharing for high-value services, preserve cost sharing for intermediate value, and increase cost sharing for low-value services. However, cost sharing for low-value services would be increased to generate a surplus sufficient to offset the costs of expanding health insurance coverage.

Strategy 1 more closely approximates current pilot studies of VBID, whereas the cost-offset alternatives may become more compelling as forces grow to limit health care spending growth while simultaneously providing insurance coverage for those currently uninsured.

### Definition of Value Strata

We benchmarked three separate tiers of value, each of which would be linked to a distinct level of cost sharing. We defined “high value” as any service with an incremental cost-effectiveness ratio (ICER) of ≤$100,000 per life-year; “intermediate value” as any service with an ICER between $100,000 per life-year and $300,000 per life-year, or with an ICER that could not be estimated because of insufficient data; and “low value” as any service with an ICER of greater than $300,000 per life-year. We chose these benchmarks because, across a wide range of plausible scenarios and assumptions, individuals in the US appear to be willing to pay at least $100,000 per life-year for health benefits but are unwilling to pay more than $300,000 per life-year for health benefits [13],[15]. These value tiers were varied in sensitivity analyses.

### Specification of How VBID Could Link Cost Sharing to Value

We reasoned that a system of linking cost-effectiveness to value should apply no cost sharing to high-value services (i.e., ≤$100,000 per life-year), because incentives to reduce the use of these services are likely to cause adverse outcomes. In contrast, we reasoned that a system linking cost sharing to value should apply substantial cost sharing to low-value services (i.e., >$300,000 per life-year), because incentives to reduce the use of these services are less likely to harm health, whereas they will reduce costs. For health services of intermediate value, either because the ICER is between $100,000 and $300,000 per life-year or because evidence is insufficient to enable value to be estimated, we assumed that prevailing levels of cost sharing would persist. We specified three tiers rather than a higher number of tiers, because this level of complexity is already accepted in the US health care system (e.g., three-tier and four-tier formularies). Although there is evidence that some therapies consumed in the US are not effective and may reduce life expectancy (e.g., PSA screening in men over 80), for our base case analyses we assume all purchased services, even low-values ones, have some positive effect on life expectancy. In sensitivity analyses, we considered the possibility that a substantial proportion of US health services are ineffective.

### Implementation of VBID in Computer Simulation

**Figure 2:**
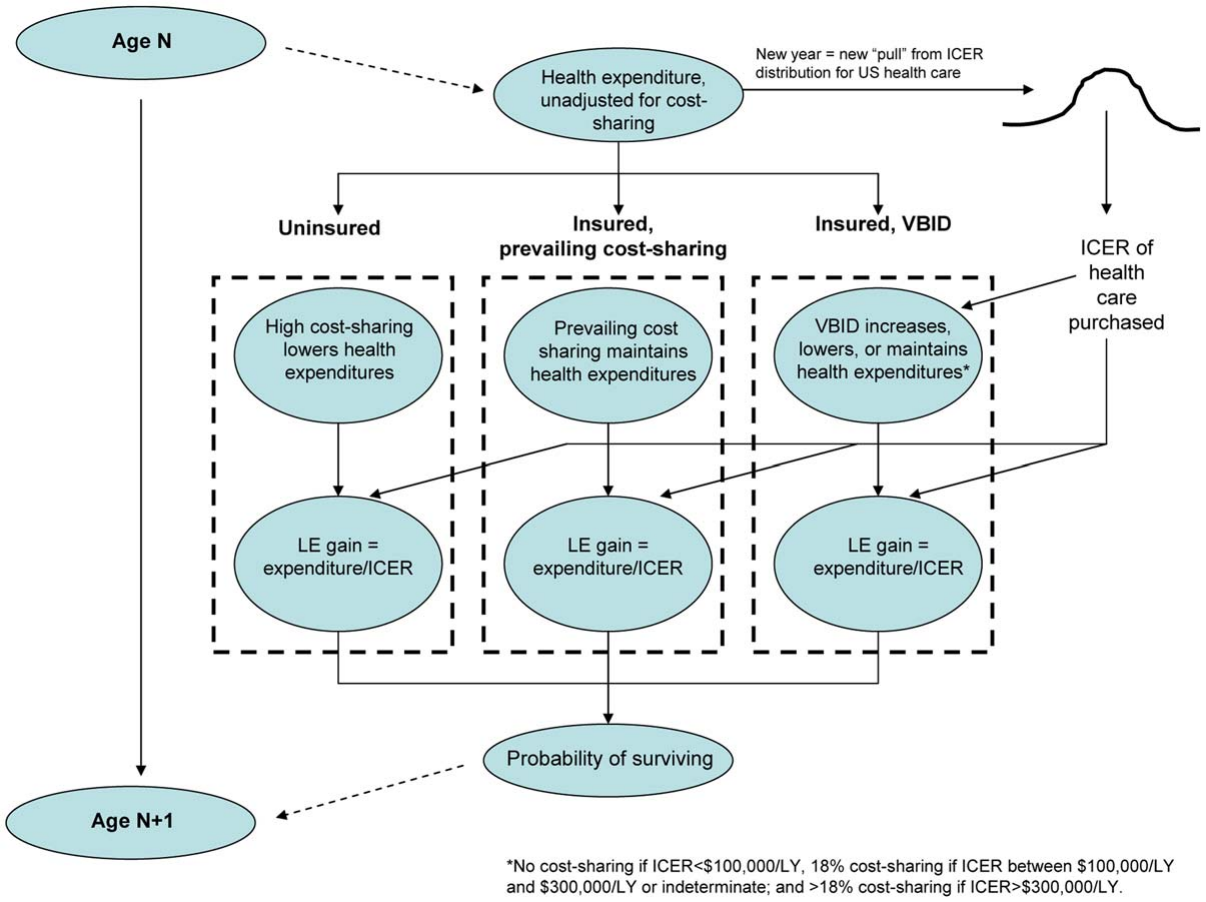
Schematic of computer simulation. Annual health expenditures vary with the amount of cost sharing. Among uninsured and among insured with prevailing cost sharing, the amount of cost sharing does not have a specified relationship with value. Among those with VBID, cost sharing falls for high-value services (which results in greater spending on these services) and rises for low-value services (which results in lesser spending on these services).

Each year, a simulated individual in our cohort would “buy” an allotment of health care based on published age-stratified health expenditure estimates [16]. When VBID is not used (Figure 2), the amount of health care “bought” was determined solely by age- and insurance-adjusted health expenditure estimates, and did not fluctuate systematically with the value of the services that were bought. When VBID was used (Figure 2), the amount of health care bought fluctuated systematically with health service value, equaling the age- and insurance-stratified expenditure multiplied by a factor reflecting the elasticity of health care demand (i.e., the extent to which health care utilization is price-dependent) with changes in cost sharing. In other words, the amount of health care bought was greater if the services were of high value (≤$100,000 per life-year), because cost sharing would be reduced; it was unchanged if the services were of intermediate value (between $100,000 and $300,000 per life-year), because cost sharing would be unchanged; and it was lower if the selected services were of low value (>$300,000 per life-year), because cost sharing would be increased.

We estimated the likelihood that services bought were of low, high, or intermediate value based on the estimated distribution of ICERs of health care services available in the US. Because this distribution is not known with certainty, we evaluated different sets of distributions, using plausibility criteria that mathematically limited the set of ICER distributions to a comparatively small group. These criteria are described in detail in Text S1, and included the requirements that health care expenditures estimated by the simulation under current circumstances must mirror current health care expenditures and health benefits. Each model run simulated one million hypothetical patients, which yielded a reproducibility of approximately 0.01 life-years. The computer simulation is described in more detail in the Text S1, and is available from the author upon request.

## Results

First, we use our mathematical model to make inferences about the value of current US health care spending. Second, we describe the results of analyses that systematically apply VBID but restrict its scope to pharmaceuticals spending. Third, we describe our results, systematically applying VBID to all health care spending regardless of service type.

### The Value of US Health Care Spending

Synthesizing evidence about US health care costs and benefits,our simulation estimated that approximately 60% of health expenditures in the US are spent on low-value services, 20% of health expenditures are for intermediate-value services, and 20% are for high-value services. Correspondingly, the vast majority (80%) of health expenditures would have cost sharing that is impacted by VBID. Even when we used the model to explore optimistic assumptions about how health expenditures are distributed (i.e., a narrow cost-effectiveness distribution, meaning that health services offered consistently favorable value), a majority of spending continued to occur on low- and intermediate-value services (52%), and a majority of spending (54%) continued to have cost sharing that is impacted by VBID.

### Applying VBID to Pharmaceuticals

Applying VBID to pharmaceutical expenditures (Table 1) increased life expectancy gain attributable to health care from 4.70 life-years to between 4.73 life-years and 4.75 life-years (a gain of 0.03–0.05 life-years). The magnitude of gain was similar (0.03 life-years) for two of the VBID design alternatives (strategies 1 and 2). Strategy 3 resulted in a greater gain (0.05 life-years) because the 0.03 life-years added by lowering copays for high-value services was supplemented by an additional 0.02 life-years from allowing more people to have access to health insurance.

**Table 1:** Life expectancy gain and health care costs with diffusion of VBID to pharmacy services.

Outcome		No VBID	VBID				
			Low-Value Copays Unchanged (Strategy 1)	Low-Value Copays Increased to Keep Spending Constant (Strategy 2)			Low-Value Copays Increased to Keep Spending Constant and Expand Insurance (Strategy 3)^d^
				*Societal Perspective* a	*Payer Perspective* b	*Patient Perspective* c	
**Life expectancy gain (life-years)**	Estimate	4.70	4.73	4.73	4.73	4.73	4.75
	Δ VBID	—	0.03	0.03	0.03	0.03	0.05
**Expenditures, per capita ($)**	Estimate	5,688	5,695	5,688	5,682	5,675	5,688
	Δ VBID	—	7	0	(6)	(13)	0
**Expenditures, national ($ billion)**	Estimate	1,654	1,656	1,654	1,652	1,650	1,654
	Δ VBID	—	2	0	(2)	(4)	0

Parentheses indicate negative numbers.

a Low-value copays set to 21%.

b Low-value copays set to 23%.

c Low-value copays set to 26%.

d Low-value copays set to 30%.

Applying VBID to pharmaceutical expenditures (Table 1) had varying effects on overall health expenditures, depending on its design. With strategy 1, annual health care spending was elevated slightly (an increase of $7 per capita, and $2 billion overall) because the increase in high-value service utilization was not balanced by a decrease in low-value service utilization. With strategy 2, low-value copays were increased as necessary to keep health expenditures constant (21%, keeping societal expenditures constant; 23%, keeping payer expenditures constant; 26%, keeping out-of-pocket patient expenditures constant), and annual health care expenditures remained unchanged or decreased slightly (to a decrement of $13 per-capita and $4 billion overall), because the increase in spending was offset by a decrease in spending on low-value services. With strategy 3, low-value copays were increased to offset expanding health insurance coverage (30%), and annual health care costs did not change, because the increases in spending on high-value services and on expanding health insurance coverage were offset by a decrease in spending on low-value services.

### Applying VBID to Other Health Services

The hypothetical scenario in which VBID was applied more broadly across health service types (not just to pharmaceuticals) resulted in substantially greater gains in life expectancy from health care (Table 2), and greater potential fluctuations in health spending. VBID increased life expectancy attributable to health care from 4.70 life-years to between 4.94 life-years and 5.14 life-years (a gain of between 0.24 life-years and 0.44 life-years). The magnitude of gain was similar (0.24–0.25 life-years) for two of the VBID design alternatives (strategies 1 and 2). Strategy 3 resulted in a greater magnitude of gain (0.44 life-years), because the 0.24 life-years added by lowering copays for high-value services was supplemented by an additional 0.20 life-years from allowing more people to have access to health insurance. When the subgroup of people without health insurance was analyzed separately, their life expectancy gain from VBID was 1.21 life-years (from 3.93 life-years to 5.14 life-years).

**Table 2:** Life expectancy gain and health care costs with diffusion of VBID to all health services.

Outcome		No VBID	VBID				
			Low-Value Copays Unchanged (Strategy 1)	Low-Value Copays Increased to Keep Spending Constant (Strategy 2)			Low-Value Copays Increased to Keep Spending Constant and Expand Insurance (Strategy 3)^d^
				*Societal Perspective* a	*Payer Perspective* b	*Patient Perspective* c	
**Life expectancy gain (life-years)**	Estimate	4.70	4.96	4.95	4.95	4.94	5.14
	Δ VBID	—	0.26	0.25	0.25	0.24	0.44
**Expenditures, per-capita ($)**	Estimate	5,688	5,760	5,688	5,623	5,555	5,688
	Δ VBID	—	72	0	(65)	(133)	0
**Expenditures, national ($ billion)**	Estimate	1,654	1,675	1,654	1,635	1,616	1,654
	Δ VBID	—	21	0	(19)	(38)	0

Parentheses indicate negative numbers.

a Low-value copays set to 21%.

b Low-value copays set to 23%.

c Low-value copays set to 26%.

d Low-value copays set to 30%.

Applying VBID more broadly across health services had varying effects on societal health expenditures depending on its design (Table 2). With strategy 1, annual health care costs were elevated (an increase of $72 per capita, and $22 billion overall), because the increase in high-value service utilization was not balanced by a decrease in low-value service utilization. With strategy 2, low-value copays were increased as necessary to keep health expenditures constant, and annual health care expenditures were unchanged or decreased slightly (up to a decrement of $170 per-capita and $48 billion overall), because the increase in spending was offset by a decrease in spending on low-value services. With strategy 3, low-value copays were increased to offset expanding health insurance, and annual health care spending did not change because the increases in spending on high-value services and on expanding health insurance coverage were offset by a decrease in spending on low-value services.

### Sensitivity Analyses

Even when we varied important assumptions in the model, VBID still could offset the incremental costs of eliminating uninsurance, and could add substantial life expectancy gains from health care (Table 3). For example, when we explicitly considered that it will never be possible to estimate the cost-effectiveness of all health services for all population subgroups, VBID increased life expectancy by a lesser but still substantial amount (from 4.70 life-years to 5.01 life-years).

**Table 3:** Sensitivity analyses of incremental life expectancy gain from health care, varying assumptions across plausible ranges.

Outcome		No VBID	VBID				
			Low-Value Copays Unchanged (Strategy 1)	Low-Value Copays Increased to Keep Spending Constant (Strategy 2)			Low-Value Copays Increased to Keep Spending Constant and Expand Insurance (Strategy 3)^a^
				*Societal Perspective* a	*Payer Perspective* a	*Patient Perspective* a	
Base case	Estimate	4.70	4.96	4.95	4.95	4.94	5.14
	Δ VBID	—	0.26	0.25	0.25	0.24	0.44
Only can estimate value for subgroup of health services (50% of expenditures)	Estimate	4.70	4.83	4.83	4.83	4.82	4.92
	Δ VBID	—	0.13	0.13	0.13	0.12	0.22
Elasticity of demand is higher (−0.39 rather than −0.31)	Estimate	4.70	5.05	5.04	5.04	5.03	5.28
	Δ VBID	—	0.35	0.34	0.34	0.33	0.58
Elasticity of demand is lower (−0.23 rather than −0.31)	Estimate	4.70	4.88	4.88	4.87	4.87	5.02
	Δ VBID	—	0.18	0.18	0.17	0.17	0.32
Some health care services have completely inelastic demand (e.g., the 31% of expenditures for inpatient care)	Estimate	4.70	4.88	4.88	4.87	4.87	5.01
	Δ VBID	—	0.18	0.18	0.17	0.17	0.31
ICER health service distribution is wider (SD 1.3 log units rather than 0.8 log units)	Estimate	4.70	4.96	4.95	4.95	4.94	5.14
	Δ VBID	—	0.26	0.25	0.25	0.24	0.44
ICER health service distribution is narrower (SD 0.3 log units rather than 0.8 log units)^b^	Estimate	4.70	4.92	4.91	4.91	4.91	5.11
	Δ VBID	—	0.22	0.21	0.21	0.21	0.41
ICER health service distribution is not normally distributed (e.g., uniform distribution)	Estimate	4.70	4.96	4.95	4.95	4.94	5.14
	Δ VBID	—	0.26	0.25	0.25	0.24	0.44
Many health services are ineffective (30% of expenditures)	Estimate	4.70	4.96	4.95	4.95	4.95	5.15
	Δ VBID	—	0.26	0.25	0.25	0.25	0.45
Many health care services are intrinsically unsuitable for copays (e.g., the 31% of expenditures for inpatient care)^c^	Estimate	4.70	5.21	5.20	5.20	5.20	5.35
	Δ VBID	—	0.51	0.50	0.50	0.50	0.65
High-value threshold is $50k/LY rather than $100k/LY	Estimate	4.70	4.93	4.92	4.92	4.91	5.12
	Δ VBID	—	0.23	0.22	0.22	0.21	0.42

a Under base case assumptions, health care confers 4.70 additional life-years and VBID can increase this benefit by up to an additional 0.44 life-years (to 5.14 life-years). Varying model assumptions changes the magnitude of this gain moderately (from 0.44 y to between 0.22 y and 0.65 y). In these analyses, copayment for low-value services is assumed to vary as needed in order to keep expenditures constant. For example, assuming greater elasticity of demand would require smaller increases in low-value copays to offset costs of expanding health insurance.

b No amount of increased cost sharing on low-value services would be sufficient to offset eliminating cost sharing on high-value services when the standard deviation is below 0.4 (because the proportion of health spending on low-value services decreases substantially). Therefore, for this particular analysis, we assumed that cost sharing was increased on both intermediate- and high-value services.

c Copays are increased on remaining services to keep overall cost sharing constant, which magnifies the impact of VBID.

## Discussion

Our results suggest that the majority of health spending in the US health care system goes toward low-value services. Therefore, broader diffusion of VBID has the potential to raise life expectancy of the US population by as much as 0.44 life-years without increasing health care costs. Notably, these benefits could occur with little or no change in the overall proportion of health expenses that are paid out-of-pocket, and without increasing the amount of cost sharing as high as current levels for tier-4 formulary drugs [3]. Limiting VBID to pharmaceuticals reduces the potential gain, but it is still meaningful (0.03 to 0.05 life-years), and comes without a corresponding increase in health care costs.

Our analysis has several important policy implications. First, implementing value-based insurance design has the potential to increase the benefits conferred by health care without increasing costs for payers, patients, or society, because decreases in cost sharing for high-value services may be offset by increases in cost sharing for low-value services. For example, health plans may increase the population benefit from a particular health service (e.g., statins) by eliminating cost sharing for some patient subgroups (e.g., those with a 10-y risk of coronary heart disease >5%), and, if necessary, increasing cost sharing for other patient subgroups (e.g., those with 10-y risk of coronary heart disease <2.5%) [17]–[19]. Second, other incentives for modulating health service utilization based on value, both on the demand side and on the supply side (Figure 1), may present similar opportunities for improving benefits while controlling costs, and should be further studied. Third, there are many known high-value services, such as colorectal cancer screening, and eliminating cost sharing for these services would have great immediate benefits (Table 4). Fourth, implementing VBID would be facilitated by knowing the incremental costs and benefits of a wider range of health services, and adds to the urgency of funding comparative effectiveness studies [11]. Fifth, there may be effective options for controlling growth in health care expenditures that are demand- rather than supply-based, and therefore would not amplify fears about non-price-based rationing of health care. Finally, cost saving from VBID has the potential to offset additional expenditures from expanding health insurance coverage, which is emerging as a policy imperative and will contribute a distinct gain in benefits from health care.

**Table 4:** Cost-effectiveness and use of selected interventions in the Medicare population.

Intervention	Cost-Effectiveness (Cost/QALY)	Implementation	Value
Influenza vaccine	Cost saving	40%–70%	High
Pneumococcus vaccine	Cost saving	55%–65%	High
Beta-blockers after myocardial infarction	<$10,000	85%	High
Mammographic screening	$10,000–$25,000	50%–70%	High
Colon cancer screening	$10,000–$25,000	35%	High
Osteoporosis screening	$10,000–$25,000	35%	High
Management of antidepressant medications	≤$30,000	40%–55%	High
Hypertensive medication	$10,000–$60,000	35%	High
Cholesterol medication as secondary prevention	$10,000–$50,000	30%	High
Implantable cardioverter-defibrillator	$30,000–$85,000	100,000 cases per year	High
Dialysis in end-stage renal disease	$50,000–$100,000	90%	High
Lung volume-reduction surgery	$100,000–$300,000	10,000–20,000 cases per year	Intermediate
Left ventricular assist devices	$500,000–$1.4 million	5,000–100,000 cases per year	Low
Positron-emission tomography in Alzheimer's disease	Dominated	50,000 cases per year	Low

Adapted from Neumann et al., 2005 [10].

QALY, quality-adjusted life year.

Because the value of certain health services (e.g., statins) will vary by patient subgroup, VBID implementation would sometimes require considering individual patient characteristics, such as particular diagnoses or indications. However, this added measure of complexity need not be insurmountable, particularly if current initiatives expand the use of health information technology. Indeed, increasing the feasibility of VBID may be a collateral benefit of rolling out health information technology. Prevailing numbers of cost sharing tiers could be maintained (i.e., 3 or 4), but they could be assigned based on value rather than cost. Electronic medical record systems (EMRs) could enable clinicians to specify the indication for a drug at the time of prescription (or could pull this information automatically from elsewhere in the EMR), much like EMRs enable clinicians to designate diagnostic codes to inform billing. Furthermore, any added complexity of considering patient-level characteristics may be offset by reduced complexity elsewhere. Provider-based cost-control measures (e.g., utilization review, pre-authorization) are complex, inefficient, and raise administrative costs, and may become less important with an increased reliance on demand-based measures such as VBID.

This is not to say that implementing VBID would be easy. First, data are currently insufficient to inform many needed analyses, and the highest-expenditure services should be priority research areas for comparative effectiveness studies. The necessary research will often require large sample sizes, numerous subgroup analyses, and consistent methods. Second, VBID would likely require a phased roll-out. For example, it could first be implemented for Medicare pharmacy benefits, second for other high-expenditure Medicare benefits (i.e., selected devices and procedures), and third to other government health benefits. If this roll-out is successful, private payers may then follow suit. Third, exceptions to value-based cost sharing decisions will often be necessary, as efficiency may sometimes need to be superseded by equity considerations. Fourth, as with any incentive system, providers or patients may try to “game” the system by over-reporting high-value services. This may result in an increased requirement for auditing some of these diagnoses. Most importantly, it will never be possible to know the value of every health care service in every setting, even with additional research. Uncertainty may exist because of biased, uncertain, or otherwise inconclusive evidence [20]. However, our sensitivity analyses suggest that substantial benefit will accrue even if only a portion of services are amenable to value estimation. Furthermore, additional funds for comparative effectiveness research will increase the numbers of services for which value estimation is possible.

It has been argued that eliminating “unnecessary” services may alone be sufficient to control health care costs, especially since as many as one-third of all health services may be unnecessary [21]. However, many of these “unnecessary” services are likely to confer small benefits for certain subgroups, and therefore it may be difficult to argue against their use based on benefit alone. For example, if a biological cancer therapy costing $100,000 per year delays tumor recurrence by one month, even if it does not prolong survival, it would be difficult to argue that it is truly “unnecessary.” In this way, VBID may offer a feasible template to modulate utilization in accord with value.

It is important to note that VBID could facilitate negotiations by payers and employers over drug prices. Drug prices used in cost-effectiveness analysis should reflect prevailing prices in the particular location or health system in which the decision will occur [22]. The incremental cost-effectiveness ratio for two drugs of similar effectiveness but different prices (e.g., a drug with a negotiated, lower price versus a similarly effective drug with a non-negotiated, higher price) would imply that the higher-priced drug has extremely low value. Therefore, the higher priced drug would be designated for a high cost sharing tier, and manufacturers are likely to negotiate aggressively in order to avoid this designation.

Our work has notable limitations. We did not consider the impact of increasing cost sharing for low-income persons, who are disproportionately impacted and would likely require copayment subsidies [23]. Our analysis does not consider the incremental costs associated with the necessary research that would be required to apply VBID more systematically. There is debate about the estimate for health care-attributable life expectancy gain that we used to anchor our analyses (4.70 y). We did not consider annual caps for deductibles or out-of-pocket spending. Because health spending is not distributed evenly, spending caps could mute the impact of VBID. However, it is possible that caps could be replaced by a more gradual reduction in cost sharing as personal expenditures increase. We analyze scenarios in which a uniform cost sharing percentage is applied across all services of similar value, and some question whether this is a realistic proposition for higher-priced services (e.g., implantable defibrillators); however, it is important to note that tiered formularies already apply uniform cost sharing percentages to drugs regardless of expense. The cost-effectiveness distribution of health services was assumed not to vary by patient age. Finally, different subgroups of drugs or services may have distinct elasticity estimates [2],[24],[25], and to keep the complexity in the model manageable, we used a uniform estimate across services. However, since our model represents a “population” of health care services, including services with above-average elasticity together with services with below-average elasticity, this heterogeneity is unlikely to undermine the validity of our results. Furthermore, sensitivity analyses showed that our results were robust across a range of elasticity assumptions that encompass much of the reported variability in elasticity by service type.

Indeed, a major methodological strength of this work is that it aims to represent the “population” of health services in the US, rather than aiming to represent only particular health services. Much like how studying a population of patients may yield more generalizable inferences than studying one or two individual patients, our approach enables us to ask policy questions about the health care system that are more generalizable and have more public health impact (e.g., should we waive copayments or deductibles for services with demonstrated high value?) than the policy questions we could ask if the model were restricted to particular services (e.g., should we waive copayments for ACE inhibitors in diabetics?). Furthermore, our “population”-based approach enables us to use mathematical modeling to make important inferences about US health care system overall (e.g., the proportion of spending on high-value services versus low-value services) that would not be possible if we considered only individual health services in isolation.

Our results suggest that society spends a majority of its health dollars on low-value services. Consequently, VBID offers the promise of saving money (by discouraging the use of low-value services) while increasing health (by encouraging the use of high-value services), and the money saved by VBID is sufficiently great to help fund universal insurance. Our results raise the broader question of whether other systematic methods of linking value to incentives may yield substantial life expectancy gains at little or no additional cost.

## Supporting Information

Text S1 Description of simulation design.
(0.14 MB DOC)

## Abbreviations

COVID: cost-offset value-based insurance design
EMR: electronic medical record system
ICER: incremental cost-effectiveness ratio
VBID: value-based insurance design
