# Supplementary material for: Can Broader Diffusion of Value-Based Insurance Design Increase Benefits from US Health Care without Increasing Costs? Evidence from a Computer Simulation Model
Source: PLoS Med. 2010 Feb 16;7(2):e1000234. doi: 10.1371/journal.pmed.1000234 (PMC2821897; doi:10.1371/journal.pmed.1000234)
Supplement: Text S1 — Description of simulation design. (0.14 MB DOC) [file pmed.1000234.s001.doc]

**Text S1. Description of Simulation Design**

***Overview***

The simulation is a probabilistic (2nd-order Monte Carlo), state-transition model with two basic states, “alive” and “dead”. People in the “alive” state accumulate 1 life-year of benefit annually, whereas people in the “dead” state do not accumulate any benefit. People in the “alive” state accrue variable levels of health care costs each year depending on age-stratified utilization levels and other factors, whereas people in the “dead” state do not accrue any health care costs. The time horizon is infinite, as it follows a birth cohort of US population until death. The simulation was originally programmed in Microsoft Excel, and was then transferred into the C programming language to enhance run speed and flexibility.

While the structure of the model is simple, the mathematical assumptions underlying the transition probabilities from alive to dead (i.e., mortality rate calculations) are more complex. In particular, there are a series of calculations that determine how mortality rates are specified, and how these specifications fluctuate with different assumptions about cost-sharing. Probabilities of death each year are based on standard, life-table estimates for age-specific mortality from the U.S. census bureau, but are then adjusted based on assumptions regarding how utilization of health care is mediated by cost-sharing.

The model first strips away all mortality benefit of modern health care, and then adds it back in individualized, measured doses; so that individuals with differing levels of cost-sharing can receive commensurately different levels of mortality benefits from that care. The advantage of this design is that it incorporates, “from the ground up,” the large degree of heterogeneity in cost-sharing in the U.S. that is attributable to demand-related factors, in particular cost-sharing and lack of insurance. It helps the simulation isolate, as the sole experimental variable, variations in cost-sharing that are mediated through demand-elasticity. In other words, some people have reduced access to care (e.g., because of uninsurance and/or very high cost-sharing) and these individuals will have less benefit from modern health care. Other people will have unrestricted cost-sharing (e.g., because they have a generous health insurance plan with no cost-sharing) and these individuals may receive more benefit from modern health care.

***Stripping away the effect of modern health care***

To strip away the effect of modern health care, we reviewed age-specific mortality rates from 1950 (our index year prior to the modern medical era) and 2003 (the most recent year with complete data available at the time of model creation).The model assumes that only a portion of the observed improvement in life expectancy between 1950 and 2003 is the result of the consumption of health care services. Data from published reports [1-5] estimate that of the observed 9.3 years of increased life expectancy, 4.7 of those are the consequence of modern healthcare. Therefore, we estimated the extent to which mortality rate decrements observed between 1950 and 2003 would need to be attenuated in order to decrease life expectancy by 4.7 years (the estimated life expectancy increase attributable to modern health care) rather than 9.3 years (the overall observed increase in life expectancy between 1950 and 2003). We determined that the mortality decrements observed between 1950 and 2003 would need to be attenuated by 53% (in other words, the improvement in mortality would need to be lowered to 47% of its observed value) to lower life expectancy by 4.7 years [1-5], based on testing different attenuation percentages in an empirical and iterative fashion until simulated life expectancy equaled the expected value. For example, the observed decrease in mortality rate between 1950 and 2003 for individuals Age 0 to 1 was 0.0248 (0.0070 subtracted from 0.0318). 53% of this mortality reduction was estimated to be attributable to health care, and therefore the mortality rate in 2003, assuming no utilization of modern health care, would be 0.0070 + 0.0248*0.53, or 0.0198. Similar calculations were made for other ages (Table A).

***Adding back the effect of modern health care, considering variations in cost-sharing mediated through demand elasticity***

People proceed through the simulation with annual probabilities of death calculated from these age-specific estimates of mortality rates in the absence of modern health care (Table A). However, for each year spent alive, each individual accrues some incremental life expectancy gain that is attributable to the benefit of health care which that individual receives. For example, if an individual uses $1,000 worth of health care during a particular year, and if that care has a value of 1 life-year per $100,000 spent; then that individual will receive a life expectancy increment of 0.01 life-years from the health care he received in that year. During the following year, that same individual may use $2000 worth of health care, but the health care used during the following year may be of different value (for example, a lower value of 1 life-year per $1,000,000 spent). The individual would then receive a life expectancy increment for 0.002 life-years that is attributable to the care received in the following year, and a total of 0.012 life-years from the $3,000 spent on health care during both years. Individuals “bank” the benefit that they buy, and it accrues at the time when they would have died in the absence of modern health care. It is important to observe that this approach assures that the additional life expectancy is not subject to competing mortality risks (otherwise we would be “double-counting” them) because these are already reflected in the estimates. In the absence of discounting, this method can be thought of as functionally equivalent to multiplying a vector of subsequent annual mortality rates by that factor which reduces life expectancy by the desired amount.

Averaged across a population, stripping away the benefit from modern care and then adding it back would be expected to have no effect on life expectancy if the policy variables were unchanged (that is, cost-sharing and uninsurance is the same as under base case scenarios). However, changing the policy variables (to evaluate hypothetical scenarios) has the potential to alter life expectancy substantially. If cost-sharing induces lower utilization of health care, or if that care is of lower value, then life expectancy would be less than for the base case scenario. If cost-sharing induces higher utilization of health care, or if that care is of higher value, then the life expectancy would be greater than for the base case scenario.

***How much care is used by individuals each year?***

Health care utilization is based on age-stratified estimates (Table B) [6], and then adjusted to reflect the variation in demand that is mediated through cost-sharing. The adjustment factor is calculated by considering the amount of cost-sharing combined with the elasticity of demand for healthcare (Table C) [7], which specifies that an increment in cost-sharing would be expected to cause a decrement in demand for health care. Individuals who have lower-than-average cost-sharing are likely to have increased health care utilization; whereas individuals who have higher-than-average cost-sharing are likely to have decreased health care utilization.

The amount of cost-sharing is an independent variable that varies with different policy scenarios. With value-based insurance design, the amount of cost-sharing will be lower when health care is of high value (which thereby increases utilization through this adjustor), and will be higher when health care is of low value (which thereby decreases utilization through this adjustor) (Table D).

Uninsurance is assumed to have the same impact as 100% cost-sharing since people are at-risk for paying full amount for care. While this may not be true in all circumstances (expectations of charity care, etc), it seems to be a reasonable approximation because the decrement in health service utilization by the uninsured is similar (35% reduced) to what would be anticipated by applying an expectation of 100% cost-sharing to the elasticity of demand for health care (Table C) [8-14].

***What is the value of health care?***

Each patient, each year, incurs a series of health expenditures; each of which elicits a new “pull” from the distribution of incremental cost-effectiveness ratios (ICER) of all U.S. healthcare services. If this pull elicits a higher ICER (meaning a greater amount spent per unit of health care benefit), then the value of the healthcare bought with that expenditure is less favorable. If this pull elicits a lower ICER (meaning a lesser amount spent per unit of health care benefit), then the value of the healthcare bought with that expenditure is more favorable. For example, suppose patient A is a 42 year-old male, who has an estimated annual health expenditure of $4,148. If a pull from the ICER distribution were to yield an ICER of $1000 per life-year for this expenditure (an uncommonly favorable value), then he will yield a life expectancy increase of 4.148 years from the healthcare that is purchased with that expenditure. On the other hand, if that same patient were to receive a pull from the ICER yielding an ICER of $1,000,000 per life-year (an uncommonly unfavorable value), then he would yield a life expectancy increase of only 0.004148 life-years (approximately one-day) from the healthcare that is purchased with that expenditure.

Annual expenditures are parsed into separate “bundles” of health services, each of which elicits a separate draw from the ICER distribution. For example, if an 82 year-old woman has an average annual healthcare expenditure of $22,630; then she would purchase 22.63 separate bundles of health services, each worth $1000, and each with a distinct pull from the ICER distribution. We partitioned annual expenditures into bundles because it has greater resemblance to the way health care is purchased in real life, and because it reduces the frequency of outliers (e.g. if an 80-year old woman received a favorable “pull” for her entire annual health expenditure, it might give her an impossibly long life expectancy). We chose $1000 as the bundle size because we empirically determined that this amount offered a good compromise between computer run speed and outlier minimization.

***Making inferences regarding possible ICER distributions***

We do not know what is the true ICER distribution of health services in the U.S., but we have reasonable estimates for (1) annual health expenditures ($5688 in 2003) [1], (2) lifetime health benefits (4.70 years in 2003) [1], and (3) a lower-bound estimate for the dispersion of ICER distribution (0.8 log units when log-transformed, based on the assumption that the distribution of ICERs in the registry (Figure A) [15] are biased towards being more narrowly dispersed than the true distribution). We sought to identify loci of distributions that satisfied all three of these known quantities (Figure B). We chose as our base case distribution that which satisfied criteria with the least dispersion (e.g. that with the most favorable median ICER), but we evaluated in sensitivity analyses alternative distributions that met these three criteria and had greater dispersion (and less favorable median ICER). Our estimation that 60% of health expenditures are likely to be low-value (>$300,000 per life-year), 20% of health expenditures are likely to be intermediate value (between $100,000 and $300,000 per life-year), and 20% of health expenditures are likely to be spent on high-value services (< $100,000 per life-year) directly follows from the distribution in this base case.

***Specifying value-based insurance design***

With policy scenarios that specified no VBID, cost-sharing would not necessarily be expected to vary with the value of the health services purchased. For example, regardless of whether the draw from the ICER distribution was $1000 per life-year or $1000000 per life-year, the cost-sharing would be the same.

However, with policy scenarios specifying VBID, cost-sharing would fluctuate systematically with the value of health services purchased [16]. If the value was more favorable than a designated threshold for acceptable value, then cost-sharing would be eliminated, whereas if the value was less favorable than a designated threshold, then cost-sharing would be increased (Table D). For example, if the designated threshold was $100,000 per life-year, and person A had a pull from the ICER distribution of $10,000 per life-year (numerically lower, and therefore, more favorable than the threshold), then for a particular health expenditure bundle of $1000 (unadjusted for the demand-elasticity effect of cost-sharing); cost-sharing would decrease from 18% to 0% for that bundle of services. As a result, this change in cost-sharing would increase person A’s utilization of those services by a factor of 6% (from Table D), increasing expenditures on that service by a proportionate amount (from $1000 to $1060). Therefore, VBID would augment the benefit from those services by 0.06 life-years, from 0.1 life- years to 0.106 life-years. However, if person A had a pull from the ICER distribution of $1,000,000 per life-year (numerically higher, and therefore less favorable, than that threshold), then cost-sharing would increase from 18% to a greater amount (for this example, 30%). As a result, person A’s utilization of that service would decrease by a factor of 10% (from Table D), decreasing expenditures on that service by a proportionate amount (from $1000 to $900). Therefore, VBID would diminish the benefit from this service by 0.0001 life- years, from 0.0010 life-years to 0.0009 life- years.

Because there is no consensus on how much VBID should increase cost-sharing for low-value services, we used the analytic machinery of the model to identify the particular quantities that would leave overall health expenditures unchanged from three different perspectives (society, payer, and individual); and we evaluated these alternatives in separate scenarios. In addition, we explored an alternative in which an increase in cost-sharing would reduce expenditures sufficiently to offset the increased societal expenditures from eliminating uninsurance. Increased societal expenditures from eliminating uninsurance were calculated based on assuming an elimination of the 35% decrement in health service utilization associated with uninsurance, together with the age-stratified prevalence of uninsurance in the United States under current circumstances (Table E) [17].

Societal expenditures included all health expenditures regardless of their source. Patient expenditures were approximated by out-of-pocket expenditures, and were calculated by multiplying total health expenditures by the percentage of cost-sharing to which those expenditures were subject (patients’ share of insurance premiums was not considered in this analysis). Payers’ expenditures were approximated by subtracting patient expenditures from societal expenditures.

***Sensitivity Analyses***

We used sensitivity analyses to test the importance of many limitations of our approach (Table F). As described in the manuscript, our results were generally robust to these sensitivity analyses, suggesting that even when we varied important assumptions in the model, VBID still could offset the incremental costs of eliminating uninsurance, and could add substantial life expectancy gains from health care. First, because it may not possible to estimate the ICER of every health care service, we asked how our results would vary assuming that a substantial proportion of health services have inestimable ICERs. Second, we varied our estimate of the elasticity of health care demand across a plausible range (-0.23 to -0.39), based on the 95% confidence interval of the source data from the RAND health insurance experiment and based on the range of elasticity estimates for different types of healthcare services. Similar ranges have also been corroborated by subsequent observational studies. Third, we explored alternative sets of thresholds for demarcating low-, intermediate-, and high-value services; first with lower thresholds (≤$50,000, $50,000 - $100,000, and >$100,000, respectively), and then with wider thresholds (≤$50,000, $50,000 - $500,000, and >$500,000, respectively). Fourth, we explored alternative ICER distributions, considering wider distributions (up to 5-times the standard deviation of the base-case distribution) and uniform distributions (differing markedly in shape from the normal distribution used in base case), while ensuring that the model remained calibrated. Fifth, we explored scenarios in which a substantial proportion of health services were assumed to be completely ineffective. Finally, we explored scenarios in which a substantial proportion of health services were deemed to be intrinsically unsuited to cost-sharing (e.g., inpatient and emergency care), and were excluded from cost-sharing of any type.

**References**

1. Braithwaite RS, Meltzer DO, King JT,Jr., Leslie D, Roberts MS (2008) What does the value of modern medicine say about the $50,000 per quality-adjusted life-year decision rule? Med Care 46(4): 349-356.
2. Bunker JP (1995) Medicine matters after all. J R Coll Physicians Lond 29(2): 105-112.
3. Bunker JP, Frazier HS, Mosteller F (1994) Improving health: Measuring effects of medical care. Milbank Q 72(2): 225-258.
4. Bunker JP (2001) The role of medical care in contributing to health improvements within societies. Int J Epidemiol 30(6): 1260-1263.
5. Mackenbach JP (1996) The contribution of medical care to mortality decline: McKeown revisited. J Clin Epidemiol 49(11): 1207-1213.
6. Meara E, White C, Cutler DM (2004) Trends in medical spending by age, 1963-2000. Health Aff (Millwood) 23(4): 176-183.
7. Keeler EB, Rolph JE (1988) The demand for episodes of treatment in the health insurance experiment. J Health Econ 7(4): 337-367.
8. Young GJ, Cohen BB (1991) Inequities in hospital care, the Massachusetts experience. Inquiry 28(3): 255-262.
9. Haas JS, Udvarhelyi IS, Morris CN, Epstein AM (1993) The effect of providing health coverage to poor uninsured pregnant women in Massachusetts. JAMA 269(1): 87-91.
10. Haas JS, Goldman L (1994) Acutely injured patients with trauma in Massachusetts: Differences in care and mortality, by insurance status. Am J Public Health 84(10): 1605-1608.
11. Haas JS, Udvarhelyi S, Epstein AM (1993) The effect of health coverage for uninsured pregnant women on maternal health and the use of cesarean section. JAMA 270(1): 61-64.
12. Canto JG, Rogers WJ, French WJ, Gore JM, Chandra NC, et al. (2000) Payer status and the utilization of hospital resources in acute myocardial infarction: A report from the national registry of myocardial infarction 2. Arch Intern Med 160(6): 817-823.
13. Roetzheim RG, Pal N, Gonzalez EC, Ferrante JM, Van Durme DJ, et al. (2000) Effects of health insurance and race on colorectal cancer treatments and outcomes. Am J Public Health 90(11): 1746-1754.
14. Piette JD, Wagner TH, Potter MB, Schillinger D (2004) Health insurance status, cost-related medication underuse, and outcomes among diabetes patients in three systems of care. Med Care 42(2): 102-109.
15. Center for the Evaluation of Value and Risk in Health. The Cost-Effectiveness Analysis Registry [https://research.tufts-nemc.org/cear/Default.aspx]. (Boston), Institute for Clinical Research and Health Policy Studies, Tufts Medical Center. Available from: [www.cearegistry.org](https://research.tufts-nemc.org/cear/default.aspx) [May 28, 2009].
16. Braithwaite RS, Rosen AB (2007) Linking cost sharing to value: An unrivaled yet unrealized public health opportunity. Ann Intern Med 146(8): 602-605.
17. U.S. Census Bureau, Statistical Abstract of the United States, 2007. Vital statistics.

**Table A. Mortality rates used in simulation.** The mortality rate in 2003 assuming no modern health care was estimated by using the simulation to identify the percentage by which the change in mortality rate between 1950 and 2003 would need to be attenuated in order to reduce life expectancy in 2003 by 4.7 years (the estimated contribution of modern health care to life expectancy, based on prior reports) [1-5]. We identified this percentage to be 53%, and therefore, we estimated that 53% of the improvement in mortality since 1950 is likely to be attributable to modern health care.

| **Age (years)** | **Mortality rate, 2003*** | **Mortality rate, 1950†** | **Change in Mortality rate between 1950 and 2003** | **Mortality rate, 2003 *assuming no modern health care*** | **Mortality decrease attributable to modern health care** |
| --- | --- | --- | --- | --- | --- |
| <1 | 0.0070 | 0.0318 | 0.0248 | **0.0198** | 0.0128 |
| 10 | 0.0002 | 0.0008 | 0.0006 | **0.0005** | 0.0003 |
| 20 | 0.0009 | 0.0013 | 0.0004 | **0.0011** | 0.0002 |
| 30 | 0.0010 | 0.0018 | 0.0008 | **0.0014** | 0.0004 |
| 40 | 0.0021 | 0.0037 | 0.0016 | **0.0029** | 0.0008 |
| 50 | 0.0044 | 0.0084 | 0.0040 | **0.0065** | 0.0021 |
| 60 | 0.0098 | 0.0186 | 0.0088 | **0.0144** | 0.0046 |
| 70 | 0.0239 | 0.0433 | 0.0194 | **0.0341** | 0.0102 |
| 80 | 0.0593 | 0.0959 | 0.0366 | **0.0786** | 0.0193 |
| 90 | 0.1540 | 0.2430 | 0.0890 | **0.2007** | 0.0467 |

**Table B. Per-capita annual expenditures for modern health care, stratified by age, unadjusted for cost-sharing-mediated changes in demand for services.** As no data were available to describe the age distribution of health expenditures in 1950, we assumed an identical age distribution to Meara et al [6]. However, because health expenditures in 1950 were only 13% of expenditures in 2003, it is unlikely that the results of our analysis would vary greatly if the age distribution in 1950 were different from our assumptions.

| **Age** | **Annual per-capita spending, 2003** | **Annual per-capita spending, 1950** | **Incremental change in spending, 1950-2003** |
| --- | --- | --- | --- |
|  |  |  |  |
| **0-5** | $2,190 | $284 | $1,905 |
| **6-64** | $4,148 | $539 | $3,609 |
| **65-74** | $13,062 | $1,698 | $11,364 |
| **75+** | $22,630 | $2,942 | $19,688 |
| **All Age Groups** | $5,698 | $741 | $4,957 |

**Table C. Impact of cost-sharing on demand for health services, based on data from the RAND Health Insurance Experiment [7].** Lowering cost-sharing increases demand for health services, whereas increasing cost-sharing decreases demand for health services. These results are consistent with those from more recent, nonrandomized studies.

| Percentage cost-sharing | Index of demand for health services  (Current = 1) |
| --- | --- |
| 0% | 1.06 |
| 18% | 1.00 |
| 20% | 0.98 |
| 30% | 0.90 |
| 40% | 0.85 |
| 50% | 0.81 |
| 100% | 0.65 |

**Table D: How VBID would influence the amount of benefit from a particular health expenditure, based on its ICER.** In our base case analysis, we used thresholds of $100,000 per life-year and $300,000 per life-year; however, we explored alternative thresholds in sensitivity analyses.

| If pull from ICER distribution is | Then value is | Then cost-sharing changes | Based on change in cost-sharing (previous column) and the elasticity of demand (Table C) utilization is: | Based on change in utilization (previous column), benefits and expenditures are: |
| --- | --- | --- | --- | --- |
| < $100,000 per life-year | High | From 18% to 0% | Increased by 6% | Increased by 6% |
| Between $100,000 and $300,000 per life-year | Intermediate | Unchanged | Unchanged | Unchanged |
| Greater than $300,000 per life-year | Low | From 18% to 30%* | Decreased by 10% | Decreased by 10% |

*We explored a wide plausible range of alternatives (20% to 50%) in sensitivity analyses. Base case assumptions were different for different scenarios. Assuming societal expenditures would stay constant required low-value cost-sharing to be set to 21%; assuming patient expenditures would remain constant required low-value cost-sharing to be set to 24%; assuming payer expenditures would remain constant required low-value cost-sharing to be set to 27%, and requiring offset of increased expenditures from eliminating uninsurance required low-value cost-sharing to be set to 30%.

**Table E. Age-stratified prevalence of uninsurance in the United States, based on data from the U.S. Census Bureau [17]**. Estimates are based on point prevalence rather than cumulative prevalence over a designated time interval (e.g., one year)

| Age group (years) | % Uninsured |
| --- | --- |
| 0-17 | 11% |
| 18-24 | 30% |
| 25-34 | 26% |
| 35-44 | 18% |
| 45-64 | 14% |
| ≥65 | <1% |

**Table F. Potential limitations in our analyses, and mitigation strategies to reduce their impact on model validity.**

| **Potential Limitation** | **Mitigation Strategy** |
| --- | --- |
| The ICER distribution of services purchased by U.S. health expenditures is unknowable | Observe that the ICER distribution is likely to be at least as wide as registry-based sample distribution, because selection bias for this registry is likely in a known direction (e.g., towards studying services perceived to be close to the value threshold, and therefore with a bias towards narrow dispersion). This observation constrains possible distributions to a tractable, evaluable subgroup. Use the lowest (most favorable) distribution as base case; test other distributions in sensitivity analyses. In addition to varying width of distribution in sensitivity analyses, also vary shape of distribution to extremes (uniform versus normal distribution) |
| There may be heterogeneity of the value of health services by age (e.g. society spends the most on elderly persons, and the value of this care might be lower) | This consideration would not greatly impact population-level results that do not involve discounting, since otherwise decreased value on high-age services would be partially compensated by increased value on low-age services. Because our base case analyses do not involve discounting, this is unlikely to undermine our results. |
| There may have been heterogeneity in growth of health service expenditures by age | Because health care spending in 1950 was only 13% (in inflation-adjusted dollars) of health care spending in 2003, even if the change was disproportionate for elderly Americans (suppose, for example, health care spending in 1950 for elderly Americans was 7% of current spending, rather than 13%, in inflation-adjusted dollars), this would have little impact on the results |
| There is great person-to-person variation in healthcare utilization. We assume that expenditures are homogeneous across identical age- and sex-strata; | This consideration would not impact population-level results because even if some people generate more expenditures or receive more benefit, these will be offset by other people receiving less expenditures and receiving less benefit, so the population-level effect will be negligible. |
| There may be heterogeneity of health benefit by utilization frequency (e.g. people who consume a large volume of health care services may be receiving services of lower value than people who receive a smaller volume, because of patient- or setting-specific factors such as high utilization regions, etc) | In the aggregate, it does not matter if low- or high-value services are cluster by person or by region; the population-level results would remain the same |
| This analysis does not discount future costs and benefits | We have chosen to not include discounting in our base case analysis because if we discounted costs we would also need to discount benefits, and discounting benefits could potentially diminish the policy relevance of this analysis. In addition, this study aims to be consistent with methods used for projecting future health expenditures (e.g. cost of eliminating uninsurance over the next 10 years), which generally are not discounted. |
| Simulation does not capture health expenditures in life-years added by healthcare | The incremental error (e.g. spending during the additional 0.02 to 0.44 life-years created by changing the cost-sharing design) is small relative to the incremental costs that incur from changing cost-sharing design over a lifetime. Therefore, this limitation is unlikely to impact our results substantially |
| It will never be possible to estimate the ICER of all health services | Sensitivity analyses: Assume that varying proportions of health services have ICERs that are unknowable. These services would have cost-sharing unchanged by VBID. (overall, has the impact of diluting any VBID implementation) |
| These analyses do not consider the incremental costs of the comparative effectiveness research that would be a prerequisite for implementing VBID | This is likely to be a second order consideration, as amounts under consideration for spending on comparative effectiveness research (e.g. $ 1.1 billion annually) are infinitesimally small compared to health care expenditures (more than $ 2 trillion annually) |
| Society may use different value thresholds than our base case analysis | Sensitivity analyses: Use thresholds of $50K and $100k |
| Simulation does not represent individual health services (e.g. ACE inhibitors for diabetics) | This is actually a strength rather than a limitation. Indeed, a major methodological innovation of this work is that it aims to represent a “population” of health services, rather than aiming to only represent particular health services. Much like studying a population of patients may yield more generalizable inferences than studying one or two individual patients, our approach enables us to ask policy questions about the health care system that are more generalizable and have more public health impact than the policy questions we could ask if the model were restricted to particular services. |
| Cost-sharing is not constant per dollar spent per individual, but rather starts out high (because of deductibles) and may end up lower (after annual out-of-pocket maximums are reached) or higher (after lifetime out-of-pocket maximums are reached). | Difficult to address in sensitivity analyses because of its complexity and heterogeneity; however, cost-sharing at particular segments of an individuals’ health consumption trajectories may be balanced by lower cost-sharing at other segments of a particular individuals’ health consumption trajectory |
| Model does not consider services that are cost-saving | Difficult to address in sensitivity analyses; however this is likely a second-order concern since few health services are really cost-saving. |
| ICER distribution assumes all services have some (albeit infinitesimally small) effectiveness, and therefore model does not represent the substantially portion of services that may be ineffective or harmful | Sensitivity analysis: Assume that some portion of spending (e.g., 30%; based on work by the Dartmouth group) is on services that are ineffective; then re-calculate range of possible ICER distributions based on the 70% of expenditures that are potentially effective, then rerun analyses. |
| Elasticity of demand for health services is likely to value by service type | 2 types of sensitivity analyses: first, vary elasticity of demand across plausible range that considers heterogeneity by service type. Second, rerun analyses assuming that a portion of health services (e.g. inpatient services) are completely inelastic. |
| Cost-sharing varies by service type | Sensitivity analyses: rerun analyses assuming that a portion of health services (eg. inpatient services) are not subject to any cost-sharing whatsoever, varying cost-sharing on the remaining services as necessary to keep overall cost-sharing per person at the estimated level. |
| Volume-averaged distribution of cost-effectiveness of health services may be narrower than the registry distribution | Sensitivity analysis: Explore distributions that are narrower than the registry-based distribution, varying the amount of cost-sharing on low-value services (and, if necessary, intermediate value services) to offset increase in spending on high-value services |
| Volume-averaged distribution of cost-effectiveness of health services may be skewed leftwards | The mean of the volume-averaged distribution is anchored by the calibration criterion reproducing the estimated costs and benefits of the healthcare system (approximately $100,000 per life-year). If the volume-averaged distribution is skewed leftward, then its median would occur rightwards of this mean value of approximately $100,000 per life-year, and therefore a majority of spending would continue to occur on services with value less favorable than $100,000 per life-year. |

**Figure A. Distribution of incremental cost-effectiveness ratios of U.S. health since 1997 based on data from the cost-effectiveness registry**. Because the registry distribution is likely to be biased (services with evidently small or great value were less likely to be selected for analysis, therefore reducing dispersion), the registry distribution was not used directly in the computer simulation. Rather, its width was assumed to be the lowest plausible value for the width of the true distribution.

**Figure B. Distributions of incremental cost-effectiveness ratios meeting calibration criteria** (under prevailing patterns of cost-sharing and uninsurance, health care confers 4.70 life-years [1], incurs annual per-capita expenditures of $5688 [1], and has a standard deviation at least as large as 0.8 log units) [15]. The graph shows normal distributions meeting these criteria, up to a dispersion of 1.8 log units.
